# EZH2 abnormalities in lymphoid malignancies: underlying mechanisms and therapeutic implications

**DOI:** 10.1186/s13045-019-0814-6

**Published:** 2019-11-21

**Authors:** Boheng Li, Wee-Joo Chng

**Affiliations:** 1 Cancer Science Institute of Singapore, National University of Singapore, Singapore, Singapore; 2 Department of Haematology-Oncology, National University Cancer Institute of Singapore, Singapore, Singapore; 3 Department of Medicine, Yong Loo Lin School of Medicine, National University of Singapore, Singapore, Singapore

**Keywords:** EZH2, T cell, B cell, Lymphoid malignancies, Lymphoma, EBV, EZH2 inhibitor

## Abstract

EZH2 is the catalytic subunit of the polycomb repressive complex 2 (PRC2), which along with other PRC2 components mediates gene expression suppression via the methylation of Histone H3 at lysine 27. Recent studies have revealed a dichotomous role of EZH2 in physiology and in the pathogenesis of cancer. While it plays an essential role in the development of the lymphoid system, its deregulation, whether due to genetic or non-genetic causes, promotes B cell- and T cell-related lymphoma or leukemia. These findings triggered a boom in the development of therapeutic EZH2 inhibitors in recent years. Here, we discuss physiologic and pathogenic function of EZH2 in lymphoid context, various internal causes of EZH2 aberrance and how EZH2 modulates lymphomagenesis through epigenetic silencing, post-translational modifications (PTMs), orchestrating with surrounding tumor micro-environment and associating with RNA or viral partners. We also summarize different strategies to directly inhibit PRC2-EZH2 or to intervene EZH2 upstream signaling.

## Background

EZH2, the enzymatic component of polycomb repressive complex 2 (PRC2), is evolutionarily conserved from drosophila to mammals. It acts as a lysine methyltransferase to catalyze methylation of histone H3 at lysine 27, thus establishing and maintaining H3K27 tri-methylation repressive marks. Apart from EZH2, the realization of histone methylation also requires facilitation from other PRC2 core components like EED and SUZ12. Besides EZH2, EZH1 as EZH2 homolog also complexes with other PRC2 subunits to trigger H3K27 tri-methylation. In physiological conditions, EZH2 only expresses in actively dividing cells and plays a vital role in cellular proliferation and differentiation. Whereas, EZH1 expresses in both dividing and differentiated cells [1].

Structurally, EZH2 protein contains a C-terminal catalytic SET domain, an adjacent CXC domain, two SANT domains, and an in-between ncRBD domain. The SET and CXC domains are required for the realization of histone methyltransferase activity [2]; the SANT domains enable EZH2 to bind to DNA for chromatin remodeling and transcriptional regulation [3]; the ncRBD domain is the binding region with non-coding RNAs.

EZH2 aberration has been seen in a wide range of cancers, including several categories of B cell and T cell lymphoid malignancies, and it is associated with poor clinical prognosis and outcomes. In most lymphoid malignant contexts, EZH2 is overexpressed and tumorigenic, yet it is repressed and acts as a tumor suppressor in T cell acute lymphocytic leukemia (ALL). In this review, we summarize the indispensable role of EZH2 in lymphoid development, the variety of mechanisms of EZH2-related oncogenesis in lymphomas, lymphoid leukemias, and myeloma, how Epstein-Barr virus (EBV) impacts EZH2 signaling as a result of virus-host interplay and effective therapeutic strategies to harness EZH2 in different cancerous contexts.

## Physiological function of EZH2 in lymphogenesis

### EZH2 in T cells

Studies in recent years highlight a crucial role of EZH2 functioning as an epigenetic repressor via the catalyzing of H3K27 tri-methylation in T cell activation [4], differentiation [4], plasticity, and lineage commitment [5, 6] by safeguarding correct downstream gene expressions. The repressive targets include key transcriptional factors which function as pivotal regulators of differentiation or self-tolerance [7–9], cytokines like interleukins, and IFN-γ [7, 10–12] as well as cell cycle-regulatory CDK inhibitors [4, 13]. EZH2 depletion in T lymphocytes disrupts genomic transcriptome and gene bivalent states [14], rendering loss-of-defense to pathogens [15], autoimmune diseases [15–17], or cell death via apoptosis [12]. Modulatory functions of EZH2 have been investigated intensively in several different subtypes and developmental phases of T lymphocyte, including T helper, T regulatory, cytotoxic T, memory T, natural killer (NK) T, and naïve T cells. Detailed findings of how EZH2 deploys H3K27 tri-methylation to regulate T cell development are summarized in Table 1 [4–18].

**Table 1:** EZH2 as epigenetic repressor in mediating T cell development

T cell subtypes	Findings	Authors
T follicular helper cell	Ezh2 deploys H3K27 tri-methylation to repress CDKN2A expression in Tfh cells, maintaining its differentiation and activation.	Li F., et al. [4]
T helper cell	EZH2 methylates H3K27 at the IL4-IL13 locus and represses IL4-IL13 expression in Th1 but not Th2 cells.	Koyanagi M., et al. [10]
T helper cell	SMAD2 and SMAD4 regulate TGF-β-mediated IL9 production via EZH2 displacement in Th9 cells.	Wang A., et al. [11]
T follicular regulatory cell	Ezh2 is required for Tfr suppressive function and transcriptional program.	Hou S., et al. [18]
Regulatory T cell	Treg cells from EZH2 −/− FOXP3+ mice display enhanced expression of target genes shared by EZH2 and FOXP3.	Sarmento OF., et al. [17]
Cytotoxic T cell and memory T cell	Terminal cytotoxic T cells display higher-level H3K27 tri-methylation than memory T cells and repression of memory T-specific genes.	Gray SM., et al. [5]
T helper cell	EZH2 keeps the correct expression of transcription factor TBX21 and GATA3 as well as cytokines like IFN-γ in Th1 and Th2 cells.	Tumes DJ., et al. [7]
Memory T cell	AKT-mediated EZH2 S21 phosphorylation attenuates EZH2 activity and enhances memory T cells to develop into T effector cells.	He S., et al. [6]
T helper cell, regulatory T cell	EZH2 epigenetically silence IFN-γ in Th2 and Treg, and IL10 in Th2. EZH2 deficiency accelerates Th cell death via apoptosis.	Zhang Y., et al. [12]
T helper cell, regulatory T cell	EZH2-deficient Th and Treg cells neither constrain T. gondii infection nor prevent autoimmune colitis.	Yang XP., et al. [15]
Regulatory T cell	Polycomb epigenetically silences FOXP3 in a KLF-dependent manner.	Xiong Y., et al. [8]
Regulatory T cell	EZH2 safeguards Treg identity by maintaining its transcriptome and thus prevents spontaneous autoimmunity.	DuPage M., et al. [16]
Regulatory T cell	FOXP3 poises its targets for repression by recruiting EZH2 upon Treg cells activation.	Arvey A., et al. [9]
NK T cell	EZH2 depletion disrupts the bivalent state at PRZF promoter and leads to the expression of non-NKT specific TCR.	Dobenecker MW., et al. [14]
Naïve CD8+ T cell	EZH2 promotes naïve CD8+ T cell proliferation by epigenetically repressing the expression of CDKN1C and CDKN2A.	Chen G., et al. [13]

Notably, the role of EZH2 in modulating T cell fate is not limited to epigenetic silencing but multifaceted. AKT-phosphorylated EZH2, recruited by TCF1, activates BCL6 expression in T follicular helper cell development [4]. In NK T cells, EZH2 as a methyltransferase directly methylates the lineage-defining transcription factor PLZF, leading to its ubiquitination and degradation, thus restricting the expansion of a subset of immune-perturbing NK T cell [19]. Plus, EZH2 is phosphorylated by the DNA-dependent protein kinase during T cell activation which then regulates DNA damage-mediated T cell apoptosis, therefore maintaining T cell genomic integrity [20]. Besides its nuclear functions of modulating gene expression and chromosomal integrity, cytoplasmic EZH2, along with other PRC2 components, directly regulates T cell receptor signaling in the course of T cell activation [21].

### EZH2 in B cells

EZH2 impacts B cell lineage commitment, maintenance, and transition through governing a repertoire of silent genomic loci. In early B cell progenitors, the level of EZH2 is abundantly seen in pro-B cells, which dropped drastically at the pre-B cell stage; EZH2-deficient B cells are developmentally impaired mainly due to a lack of immunoglobulin μ chain production. This indicates that EZH2 is involved in the checkpoint mechanisms controlling the pro-B to pre-B transition [22]. Germinal center (GC), the central factory of the humoral immune response, refers to the specialized microstructure within secondary lymphoid tissues formed upon infection or immunization. It is where maturing B cells undergo somatic mutations of B cell receptor genes, thereby producing a series of sublines. The long-lived antibody-secreting plasma cells and durable memory B cells are selected from these clones based on their antigen-binding affinity which ensure potent immune response against pathogens [23]. EZH2 also has profound influences on preserving a well-ordered GC development process. When resting B cells enters into cell cycle for activation, H3K27 tri-methylation marks increase noticeably along with other histone H3 methylation forms to orchestrate gene expressions and establish B cell identity at different developmental stages [24, 25]. In this process, EZH2 silences anti-proliferative genes, including CDKN family cell cycle-related tumor suppressors (CDKN1A, CDKN1B, and CDKN2A) [26, 27], through the conferment of H3K27 tri-methylation marks, and helps establish bivalent chromatin at key regulatory loci to transiently repress GC B cell differentiation [28], making provisions for antibody diversification, affinity maturation [29], and secretion [30].

## EZH2 deregulation

### Genetic and epigenetic abnormalities of EZH2

Among all categories of lymphomas and lymphoid leukemias, gain-of-function somatic mutations of EZH2 were mostly detected in GC-derived diffuse large B cell lymphoma (DLBCL) and follicular lymphoma (FL) [31–34]. In these malignancies, EZH2 mutations are causally linked to disease pathogenesis and transformation [28, 31]. This corresponds to the physiological role of wild-type EZH2 in GC formation by which EZH2 imprints repressive marks on proliferation-checkpoint genes and terminal-differentiation genes to temporarily maintain GC centroblast phenotypes in order to allow for important immune events like immunoglobin affinity maturation [27, 28]. Heterozygous mutation on tyrosine 641 (Y641H, Y641S, Y641C, Y641F, and Y641N) is the most widely occurred EZH2 activating mutation in GC-originated non-Hodgkin lymphoma (NHL), accounting for approximately 22% of GC-DLBCL and FL [35]. This point mutation is located within the catalytic SET domain of EZH2. It is associated with overall increased H3K27 tri-methylation level, suppressed expression of PRC2 target genes [35–38], and a global redistribution of the tri-methylation mark [39]. Collaborations between wild-type and Y641 single allele-mutated EZH2 for intensified conversion to H3K27 tri-methylation form have been observed that wild-type EZH2 displays greatest catalytic efficiency for H3K27 mono-methylation but decreased activity for subsequent di- and tri-methylation, and in contrast, Y641-mutated EZH2 exhibits limited efficiency to catalyze H3K27 mono-methylation but augmented catalytic activity for subsequent reactions [40]. The Y641-mutated EZH2 is resistant to JAK2/BTRC-mediated degradation and is thus more stable than wild-type EZH2 [41]. Besides, EZH2 A677G activating mutation was also identified in GC-NHL, which is related to enhanced H3K27 tri-methylation [35, 42]. In contrast, loss-of-function and non-sense EZH2 mutations were found in ALL, along with deletions and mutations of other PRC2 subunits [43–47]. In addition, missense EZH2 point mutations were also identified in a few Burkitt lymphoma cases with functions not fully determined [48]. Frequencies of EZH2 mutations in different malignant contexts have been summarized before [37, 49].

Besides, genetic lesions of EZH2, including copy number amplification, chromosomal gain or loss, and DNA hypermethylation, were also identified. In DLBCL, a total of 7.7% of cases harbored chromosomal gain and 2.7% had a chromosomal loss of EZH2 [50]. Moreover, 24% of FL and 39% of transformed FL (histologically transformed from FL to high-grade and aggressive type of NHL) patients had gain or amplification of EZH2 at chromosome 7q [31]. Promoter hypermethylation of EZH2 gene body was found in pediatric ALL, with 60% of patients displaying higher methylation at EZH2 CpG island than healthy donors [44].

### Non-genetic causes of abnormal EZH2 expression

Dysregulated signaling pathways also cause abnormal EZH2 expression. C-MYC (hereafter MYC) overexpression or rearrangements have been uncovered in different types of aggressive B cell or T cell lymphomas, and the MYC-miRNA-EZH2 oncogenic axis or loop has been demonstrated in several reports, including ours [51–54]. Mechanistically, MYC upregulates EZH2 through repression of the EZH2-targeting miRNAs (miR-26a, miR-26b, miR-101) [52–54], and EZH2 could induce MYC expression in reverse via inhibition of the MYC-targeting miR-494 [54]. In multiple myeloma (MM), downregulated miR-138 brings about the upregulation of EZH2 target gene [55]. Besides, the NF-ΚB family transcription factors REL [56] and IL-6 [57] are critical EZH2 activators that upregulate EZH2 expression level in T-ALL or MM cells. In addition, proteolytic system deregulation could stabilize EZH2 protein and lead to its overexpression. As stated above, the recurrent Y641 EZH2 mutation in DLBCL and FL prevents JAK2-mediated phosphorylation and BTRC-mediated proteasomal degradation, leading to stabilization of EZH2 expression [41]. In natural killer/T cell lymphoma (NKTL), overexpression of MELK kinase phosphorylates and stabilizes EZH2 via modulation of EZH2 ubiquitination status [58]. Moreover, in chronic lymphocytic leukemia, microenvironment-induced NF-ΚB regulates genes associated with cell cycle, reactive oxygen species (ROS), and protein stability, resulting in suppression of RB and induction of EZH2 [59].

## EZH2 mediates lymphoid oncogenesis

### As epigenetic modifier

The most commonly observed function of EZH2 as a modulator of tumorigenesis is through epigenetic modification at histone H3 lysine 27. As target gene clusters of EZH2 such as the SOX genes and KIF family [60] are essential to maintain the developmental integrity of T cells and B cells, transcriptional impairment of these genes by EZH2 deregulation in lymphoid cells may thus give rise to malignant progression. In B cell lymphomas, overexpressed EZH2 hypermethylates the promoter of FAS-AS1 lncRNA and represses the FAS-AS1 expression, leading to impaired FAS-mediated apoptosis [61]. EZH2 activation also represses the tumor-suppressive BLIMP1 in GC-B cell lymphoma [29]. In B cell ALL, up-regulated EZH2 was shown to transcriptionally hold back the expression of tumor suppressor PTEN and P21 [62]. In MM, a genome-wide profiling of H3K27 tri-methylation identified a positive correlation between PRC2-mediated gene silencing and disease progression or poor prognosis [63]. A number of EZH2 target genes relating to MM pathogenesis and progression have been characterized; for example, RBPMS [55], which confers chemo-resistance to MM cells. In T-ALL, deficiency of EZH2 and H3K27 tri-methylation results in activation of IGF1, which relates to leukemia stem cell property [64], and CRIP2, which represses apoptosis [65], as well as genes involved in the cytokine signaling pathways and early hematopoietic transcriptional regulators [66]. Importantly, EZH2 may transcriptionally coordinate with other oncogenic regulators. Aberrantly activated NOTCH1 in T-ALL binds to the promoter of HES1, a critical mediator of leukemogenesis [67], and forces considerable eviction of EZH2 and other PRC2 subunits as well as loss of H3K27 tri-methylation repressive marks [46], reinforcing the activation of HES1. Defectively mutated RB fails to recruit EZH2 to diverse repeat DNA sequences as RB-EZH2 complex, disperses H3K27 tri-methylation from these genomic locations and permits repeat expression, thus conferring lymphoma susceptibilities [68].

In recent years, the non-canonical role of EZH2 as a transcriptional co-activator has been demonstrated in several types of cancer [52, 69–72]. Our team has reported that in the NKTL oncogenic role of EZH2 is independent of its methyltransferase activity [52]; the Y244-phosphorylated EZH2 binds to RNA POLII and trans-activates a set of genes associated with proliferation, cell cycle, DNA replication, stemness, and invasiveness [69].

### Associating with long non-coding RNAs

In recent decades, long non-coding RNAs (lncRNAs) have emerged as an indispensable regulator in almost every aspect of biology, and mounting evidence reveals that lncRNAs play an important role in tumorigenesis by modulating chromatin organization, transcription and post-transcriptional regulation [73]. The interplay between EZH2 and lncRNA is noticeable when it comes to EZH2-mediated oncogenesis. HOTAIR was originally demonstrated as a modular scaffold that provides binding surfaces to tether EZH2 with other histone-modifying complexes [74]. Later studies in several cancerous contexts revealed that HOTAIR modulates gene repression [75–80] or associates with other oncogenic players [81, 82] in both PRC2-dependent and independent manners to promote malignant phenotypes. In DLBCL, upregulated HOTAIR is validated as an independent indicator of poor prognosis [83] and thought as one of the possible mechanisms for inducing H3K27 tri-methylation via EZH2-related PRC2 activation [84]. MALAT1, another EZH2-binding lncRNA, regulates cancer cell metastasis [85–88] and chemo-resistance [89] in different solid tumors via EZH2-associated mechanisms. In mantle cell lymphoma, elevated MALAT1 expression correlates with poor prognosis and reduces overall survival, and knockdown of MALAT1 results in an enhanced level of P21 and P27 through the modulation of EZH2 [90]. In T and NK cell lymphoma, the EZH2-binding MALAT1 is highly expressed and linked to poor prognosis [91]. In addition, ANRIL, frequently overexpressed and tumorigenic in a panel of cancers [92–95], forms a ternary complex with EZH2 and P65, and epigenetically marks and represses P21/CDKN1A transcription in adult T cell leukemia [96].

### Regulated by phosphorylation

Over the past few years, various forms of EZH2 post-translational modifications (PTMs) have been uncovered. These include phosphorylation, acetylation, ubiquitination, and O-GlcNAcylation, all of which confer marked biological impacts to EZH2 functions. Phosphorylation is the most well-studied EZH2 PTM, and EZH2 phosphorylation at multiple distinct residues yields critical influences or are even vital to EZH2-related lymphoid oncogenesis. The firstly reported EZH2 S21 phosphorylation, observed in CD138+ bone marrow cells of MM patients, endows cell-adhesion-mediated drug resistance to MM cells through H3K27 hypomethylation-induced activation of IGF1 [97]. As stated earlier, JAK2- and MELK-directed EZH2 phosphorylation each modulates stability and proteasome-mediated degradation of EZH2 in DLBCL/FL and NKTL, respectively [41, 58]. Moreover, EZH2 phosphorylation by JAK3 in NKTL, as we reported previously, functionally switch the role of EZH2 from PRC2-associated epigenetic repressor to PRC2-independent transcriptional activator [69]. CDK1-mediated EZH2 phosphorylation, initially demonstrated as a key mechanism that regulates cell cycle and differentiation in embryonic stem cells [98–100], is crucial for EZH2 binding to tumorigenic HOTAIR [98] and MALAT1 [90].

### Modulating tumor microenvironment

Apart from mediating tumorigenicity in cancer cells, EZH2 also has a role in the regulation of tumor microenvironment. PRC2 is activated in hematopoietic bone marrow resident cells besides tumor cells in DLBCL patients and correlates with poor outcomes [101]. In cutaneous anaplastic T cell lymphoma, EZH2 not only boosts lymphoma cell survival but also represses CXCL10 to hinder the recruitment of effector CD4+ and CD8+ T cells into the microenvironment, suggesting EZH2 possesses dual roles here [102]. In DLBCL, the survival-favoring EZH2 Y641 mutation is also a genetic mechanism underlying MHC-II deficiency, which leads to escape from immune surveillance and inferior prognosis [103]. In CLL-associated fibroblasts, NF-ΚB-activated EZH2 antagonizes a subset of senescence-associated genes to promote tumorigenesis [59].

### Connected with EBV infections

EBV is one of the human herpes virus that was firstly observed in Burkitt lymphoma cells [104]. It mostly transmits via saliva and oral contacts. Primary infection of EBV is widespread and tends to be latent and asymptomatic in most individuals. However, as lymphocytes are the major host cells for the EBV natural life cycle, EBV infection is closely associated with onsets of several types of lymphomas and lymphocytic leukemias both in the immunocompetent and immunosuppressive states [105, 106].

The life cycle of EBV is regulated by chromatin modifiers which epigenetically switch EBV protein expression on and off. EZH2 is one of these modifiers involved in the maintenance of EBV latency and reactivation acting by conferring H3K27 tri-methylation marks to EBV-associated genes [107]. Of note, EBV-encoded proteins also utilize or synergize with EZH2 to silence tumor suppressor genes to drive B cell lymphomagenesis. The viral oncoprotein LMP-1 induces the recruitment of inhibitory protein complexes containing EZH2 to the promoter of tumor suppressor gene DOK1, resulting in H3K27 tri-methylation-mediated repression [108]. LMP-1-dependent activation of JNK-1 facilitates the recruitment of P73 transcription factor to the promoter of ΔNP73α, a strong antagonist of P53, in accord with the displacement of EZH2 within the same locus [109]. Together with EZH2, EBV nuclear antigens EBNA3A and EBNA3C co-silence CDNK2A/B loci, which encode P14^ARF^, P15^INK4B^, and P16^INK4A^ as well as the BIM enhancer hub, through inducing chromatin looping, disrupting physical interactions between the promoters or depositing H3K27 tri-methylation silencing mark [110, 111]. Importantly, EBV viral gene products may indirectly affect EZH2 expression by modulating EZH2 upstream gene NF-ΚB via multifaceted mechanisms. EBV viral proteins regulate NF-ΚB signaling cascade dynamics within each stage of entry-latency-host lysis infection cycle through mimicking host proteins, binding to host receptors or other forms of interplay [112].

## Therapeutic interventions to EZH2 signaling

### EZH2 inhibitors

The adenosine homolog DZnep, originally developed as an anti-HIV1 agent [113] and S-adenosylhomocysteine hydrolase inhibitor [114], was the first drug used to inhibit EZH2 expression [115]. DZnep effectively depletes cellular levels of EZH2, inhibits tri-methylation of Histone H3 at lysine 27, and induces cell apoptosis in a variety of cancerous contexts [115–118], including Burkitt lymphoma [119], ALL [120], and NKTL [52]. However, Dznep is a global histone methylation inhibitor rather than a H3K27-specific one [121].

Since the year 2012, several serials of specific EZH2 inhibitors, which compete with S-adenosylmethionine for the binding to EZH2 enzymatic SET domain, have been reported (Table 2). These inhibitors could suppress H3K27 tri-methylation, reactivate silenced PRC2 target genes and demonstrated effectiveness in inhibition of cell survival in GC-derived B cell lymphomas harboring EZH2-activating mutations amid several types of lymphoid malignancies [35, 122–140].

**Table 2:** Specific EZH2 inhibitors in lymphoid malignancies

Inhibitor(s)	Malignancies	Authors
GSK126	EZH2-mutant and wild-type GC-DLBCL, FL, ABC-DLBCL, T-ALL, MCL, ATLL, CTCL, MM	Xu L., et al. [122]; Lue JK., et al. [123]; Adamik J., et al. [124]; Zeng D., et al. [125]; Ott HM., et al. [126]; McCabe MT., et al. [35]
GSK343	MM	Ezponda T., et al. [127]
EI1	EZH2-mutant GC-DLBCL	Qi W., et al. [128]
EPZ-6438	EZH2-mutant and wild-type GC-DLBCL, MM	Herviou L., et al. [129]; Dimopoulos K., et al. [130]; Brach D., et al. [131]; Knutson SK., et al. [132]
EPZ005687	EZH2-mutant and wild-type GC-DLBCL	Knutson SK., et al. [133]
EPZ011989	EZH2-mutant GC-DLBCL	Campbell JE., et al. [134]
EBI-2511	EZH2-mutant GC-DLBCL	Lu B., et al. [135]
ZLD10A	EZH2-mutant GC-DLBCL	Song X., et al. [136]
DCE_42/254	EZH2-mutant and wild-type GC-DLBCL	Wu Y., et al. [137]
CPI-1205	EZH2-mutant GC-DLBCL	Vaswani RG., et al. [138]
Tetramethyl-piperidinyl Benzamides	EZH2-mutant GC-DLBCL	Nasveschuk CG., et al. [139]

*Abbreviations*: *GC-DLBCL* germinal center diffuse large B cell lymphoma, *ABC-DLBCL* activated B cell-like diffuse large B cell lymphoma, *FL* follicular lymphoma, *T-ALL* T cell acute lymphoblastic leukemia, *CTCL* cutaneous T cell lymphoma, *MCL* mantel cell lymphoma, *ATLL* adult T cell leukemia/lymphoma, *MM* multiple myeloma

Among these inhibitors, EPZ-6438 (Tazemetostat) is a representative that has already entered clinical trial phase I/II for the treatment of multiple malignancies with EZH2 aberrance, including GC-derived and other types of B cell lymphoma (NCT03010982, NCT03028103, NCT01897571, and NCT02875548) [141].

As both EZH1-containing PRC2 and EZH2-containing PRC2 complexes contribute to the maintenance of H3K27 tri-methylation marks, dual inhibitors of EZH1/2, like UNC1999, were developed to target the homologous enzymatic SET domain [142, 143]. In addition, a group of Tanshindiol compounds, major active components of the root of *Salvia miltiorrhiza*, also displayed inhibitory effects on EZH2 methyltransferase activity [144]*.*

Differential response to EZH2 small-molecule inhibitors like EI1 and GSK126 were observed in GC B cell lymphoma cell lines, owing to the activation of IGF-1R, MEK, and PI3K pathways [145], MLL1-P300/CBP-directed H3K27 acetylation gain [146] as well as secondary EZH2 mutations in wild-type and mutant EZH2 alleles [147, 148].

### Inhibiting EED

**Fig. 1:**
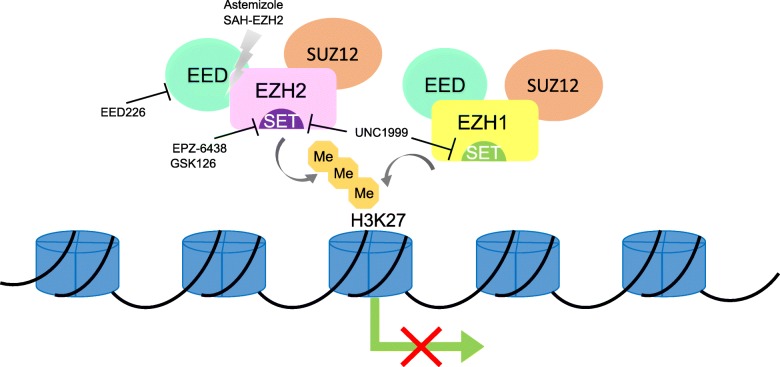
Schematic diagram showing agents specifically inhibit EZH1/2 or EED

Another strategy to harness H3K27 hypermethylation for therapeutics is to target the regulatory PRC2 subunit EED or EED-EZH2 interaction. SAH-EZH2, a peptide inhibitor which dissociates bound EZH2-EED, degrades EZH2, diminishes H3K27 tri-methylation, and inhibits cell proliferation in MLL-AF9 cells [149]. Treatment with this peptide in NKTL cells also inhibits H3K27 tri-methylation, but leaves EZH2 expression intact [69]. Astemizole, originally a second-generation anti-histamine, was withdrawn due to its potential to cause arrhythmia. Later studies uncovered that Astemizole could disrupt EZH2-EED interaction and arrest cell proliferation in some B cell lymphoma cell lines [150]. Moreover, several selective EED inhibitors binding to the H3K27 tri-methylation pocket on EED have been synthesized [151]. Among them, EED226 could induce a conformational change upon its binding to EED and demonstrated sustained sustain tumor regression effects in EZH2-mutant preclinical DLBCL model [151–153]. Therapeutic strategies for targeting EZH1/2 or EED are depicted in Fig. 1.

### Inhibiting EZH2 upstream signaling

**Fig. 2:**
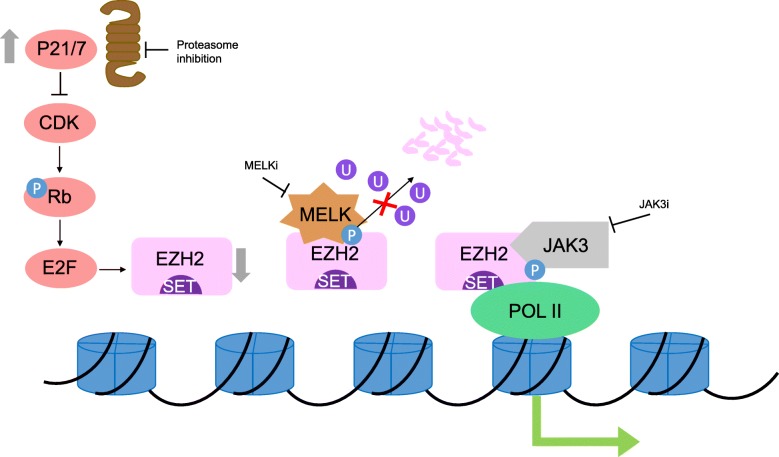
Schematic diagram showing strategies of inhibiting EZH2 upstream signaling

Apart from directly targeting the PRC2 complex, recent studies on EZH2 signaling pathways, including ours, also implied potentials of targeting EZH2 upstream factors for therapeutic purposes (Fig. 2). Proteasome inhibitors Bortezomib and Carfilzomib block proteasome-mediated degradation of CDK inhibitors P21 and P27, therefore depleting EZH2 expression level via CDK-RB-E2F axis in MM [55, 154]. In NKTL, selective JAK3 inhibitor PF-956980 removes EZH2-Y244 phosphorylation and thus obliterates oncogenic and downstream activating function of EZH2 [69]. MELK inhibition in this malignancy effectively diminishes EZH2 level by eliminating EZH2 S220 phosphorylation and promoting associated K222 ubiquitination [58].

### Combinatorial targeting with other chemotherapeutic agents

In recent years, therapeutics combining individual EZH2 inhibitor with other drugs have been explored. Synergistic or re-sensitizing effects between EZH2 inhibitors and traditional cytotoxic anti-cancer drugs have been seen [155, 156]. In view of the abundantly present epigenetic abnormalities of H3K27 tri-methylation and histone acetylation, a couple of studies have combined EZH2 inhibitors with different HDAC inhibitors for the treatment in MM [157, 158] and B cell lymphoma cells [54, 123, 159, 160], and favorable therapeutic coordination were observed. Dual-targeting of MYC and EZH2 has been employed to disrupt MYC-EZH2 oncogenic axis in aggressive B cell lymphoma cells [51]. In addition, EZH2 inhibition has also been combined with inhibition of REL, a transcriptional activator of EZH2 expression [56]. Details of combinational regimens are summarized in Table 3.

**Table 3:** Combining EZH2 inhibitors with other chemotherapy agents in lymphoid malignancies

Treatment regimen(s)	Malignancies	Authors
GSK126 and Etoposide	GC-DLBCL, BL	Smonskey M., et al. [155]
GSK126 and Pentoxifylline	MM, T-ALL	Neo WH., et al. [56]
GSK126/EPZ-6438/DZnep and Panobinostat	MM, MCL	Kalushkova A., et al. [158]; Fiskus W., et al. [117]; Fiskus W., et al. [159]; Harding T., et al. [157]
DZnep and JQ1	BL, MCL, GC-DLBCL	Zhao X., et al. [51]
DZnep and Daunoblastine	T-ALL	D’Angelo V., et al. [156]
UNC1999 and Bortezomib/Carfilzomib	MM	Rizq O., et al. [154]
DZnep and Vorinostat	MCL, BL	Zhang X., et al. [54]
GSK126/Dznep and ACY-957/1044	EZH2-mutant GC-DLBCL	Johnson DP., et al. [160]

*Abbreviations*: *GC-DLBCL* germinal center diffuse large B cell lymphoma, *DLBCL* diffuse large B cell lymphoma; *T-ALL* T cell acute lymphoblastic leukemia, *MCL* mantel cell lymphoma, *MM* multiple myeloma, *BL* Burkitt lymphoma

## Conclusions and future directions

The research evidence accumulated in this review demonstrates an indispensable role of physiological EZH2 in mediating normal B cell and T cell lymphogenesis and reveals how deregulated EZH2 modulates pathogenesis of lymphoid malignancies. Major causes dictating EZH2 aberrance are genetic abnormalities including somatic mutations, chromosomal gain/loss, and promoter hypermethylation as well as translational and post-translational causes via multiple signaling pathways. Pathogenic EZH2 modulates lymphoid oncogenesis by epigenetic repression of tumor suppressors, orchestrating with lncRNAs, site-specific PTMs, affecting microenvironment and EBV-host interplay. In recent years, an emerging interest in investigating how EZH2 assists tumor cells to escape immune surveillance has developed, and more efforts are required in future studies to clarify the exact role of EZH2 in facilitating a tumorigenic microenvironment in different types of lymphoid malignancies.

In the recent decade, a couple of strategies have been adopted to harness EZH2 deregulation for therapeutic intervention. Although the oncogenic mechanisms of EZH2 have already been uncovered by a number of in-depth studies, PRC2-based EZH2 therapeutics still have a long way to go. Dozens of chemotherapeutic agents have been developed to target the EZH2 enzymatic SET domain for therapeutics; yet, for most of these drugs, satisfactory effectiveness was only seen in B cell lymphoma cell lines or xenografts with EZH2 gain-of-function mutations. Although several compounds of EZH2-SET inhibitors have entered into clinical trials, some have already failed in phase I at least partly due to the negative mediation of anti-tumor immunity [161]. Development of EZH1/2 inhibitors and EED inhibitors represents a big leap, as these agents effectively overcome chemo-resistance of EZH2-SET inhibitors GSK126 and EPZ-6438 in DLBCL [145].

Due to the fact that none of the commercialized EZH2-specific inhibitors was able to bring down EZH2-mediated lymphomagenesis in NKTL, JAK3, or MELK inhibition has been exploited for dual-targeting of the kinase and EZH2 as an alternative strategy [52, 58, 69]. Future studies are still required to precisely deplete tumorigenic EZH2. Given that EBV infections manifest in all cases of NKTL as well as in some cases of Burkitt lymphoma and DLBCL [105], anti-EBV treatment may well complement EZH2-based therapeutics. Studies determining whether combining antivirals and EZH2 inhibitors could yield synergism are therefore needed.

## Data Availability

Data sharing is not applicable to this review article as no datasets were analyzed.

## Abbreviations

ALL: Acute lymphocytic leukemia
DLBCL: Diffuse large B cell lymphoma
EBV: Epstein-Barr virus
FL: Follicular lymphoma
GC: Germinal center
LncRNAs: Long non-coding RNAs
MM: Multiple myelomas
NHL: Non-Hodgkin lymphoma
NK: Natural killer
NKTL: Natural killer/T cell lymphoma
PRC2: Polycomb repressive complex 2
PTMs: Post-translational modifications
ROS: Reactive oxygen species
